# Governing the Interlinkages between the Sustainable Development Goals: Approaches to Attain Policy Integration

**DOI:** 10.1002/gch2.201700036

**Published:** 2017-11-13

**Authors:** Jale Tosun, Julia Leininger

**Affiliations:** ^1^ Institute of Political Science Heidelberg University Bergheimer Straße 58 69115 Heidelberg Germany; ^2^ German Development Institute Tulpenfeld 6 53113 Bonn Germany

**Keywords:** governance, implementation, policy change, policy coherence, policy integration, Sustainable Development Goals (SDGs)

## Abstract

The Sustainable Development Goals (SDGs) of 2015 form a universal and integrated policy agenda to be realized over the next 15 years. One of the targets is the attainment of policy coherence for sustainable development, which requires the individual goals to become interlinked. This article's main research interest lies in assessing how national governments and their competent ministries interpret and strive to implement the target of policy coherence for sustainable development. Drawing on the Voluntary National Reviews submitted in 2016 and 2017 by six countries, this study shows that at the national level, the links among the different goals and the idea of policy integration are subject to divergent interpretations. The differences observed do not stem from the interlinkages of the SDGs as defined by the United Nations, neither do they result from different levels of income or degree of political centralization. Instead, the respective domestic policy‐making processes are likely to explain the implementation strategies adopted by the individual states. For example, the implementation approach adopted by the government of Turkey suggests that path‐dependency is critical, whereas the Colombian approach consists of defining new policy measures and institutional arrangements.

## 1 Introduction

The 2015 United Nations (UN) Sustainable Development Summit held in New York once more demonstrated that governments all around the world have recognized the need for a global process of social and political adaptation to limits posed by the natural resource base. The limitations that both, the member states both the member states of the Organization for Economic Cooperation and Development (OECD) and developing countries face include threats to food, water, and energy security as well as health and the societies' capacity to mitigate and adapt to climate change.^1^, ^2^ These challenges have been recognized since 1972 when the first UN Conference on the Human Environment was held in Stockholm. Ever since, the attainment of sustainable development has been on the agenda of international organizations and national governments alike, but the policy actions needed failed because they were not sufficiently ambitious or not implemented adequately.^3^, ^4^ A first change to a global and outcome‐oriented policy framework for development took place with the adoption of the eight Millennium Development Goals (MDGs). The MDGs guided an era of development policy in the period between 2000 and 2015.^3^, ^5^, ^6^

However, while the MDGs outlined an agenda that attempted to align development with sustainability, UN member states set a particular focus on sustainable development when adopting the 2030 Agenda for Sustainable Development in September 2015 (e.g., ref. 7, see Note 1). The 2030 Agenda, of which the Sustainable Development Goals (SDGs) form the core, is a global policy framework that aims at reducing poverty and inequality as well as strengthening sustainable economic and social development by means of establishing a collaborative partnership between state and non‐state actors in the global South and North. Based on the principle of universalism, all UN member states—be it OECD or developing countries—committed themselves to implementing the 2030 Agenda. Another outstanding feature of the SDGs is that they are “integrated and indivisible.”^8^ From this, it follows that the synergies and trade‐offs between individual SDGs require a systematic analysis.^9^, ^10^

Prior studies focused on the quality of interlinkages of SDGs, for instance, whether they influenced each other positively or negatively.^2^, ^11^, ^12^ This study assesses the SDGs with a criterion formulated in Target 17.14, namely to enhance policy coherence for sustainable development. There exists an insightful body of policy studies that examines what policy coherence is and when it is likely to be attained (for an overview, see ref. 13). In a nutshell policy coherence “implies that various policies go together because they share a set of ideas or objectives.”^14^ This definition of policy coherence fits perfectly with the structure of the SDGs, which correspond to a “network of goals.”^11^, ^15^ Policy coherence can be attained in different ways such as adopting policy measures that help to mutually realize the goals of two or more policy areas or by assessing the effects the policy goals in one area can have on the attainment of the goals in another area.^16^ We are interested in how the SDGs related to food, water, and energy security as well as health and societies' capacity to mitigate and adapt to climate change relate to each other—this is the first research question guiding this study. At this stage of our analysis, we are interested in the international guidelines, which we expect to inform the approaches adopted by national governments to implement the SDGs.

The next research dimension takes into consideration the fact that the SDGs are only effective when they are implemented by the UN member states.^7^ From this perspective, the attainment of policy coherence does not only depend on how the SDGs as defined by the UN relate to each other. Perhaps more crucially, the attainment of policy coherence depends on how the competent national authorities interpret Target 17.14 and how they relate the five SDGs of interest to this study to each other. We expect variations between the individual countries in the approaches they adopt to consider the interlinkages between different SDGs. Therefore, the second and third research questions read as follows: How do national governments interpret the call for enhancing policy coherence for sustainable development? What are the implications of the respective national approaches for attaining the SDGs related to climate change, energy, agro‐food, health, and water provision? The answers to these questions have important implications for the study of policy coherence as well as policy‐making in practice.

The remainder of this article unfolds as follows. First, we outline the launch of the SDGs and explain what goals they established. In doing so, we concentrate on the five thematic areas identified above in order to narrow down the analysis. Then, we introduce our underlying theoretical reasoning, before moving on to the empirical analysis. The last part summarizes the main insights and offers some concluding remarks and suggestions for future research.

## 2 Sustainable Development Goals: A Call for Policy Integration

The SDG process roughly started in 2006 at the UN General Assembly, when South African President Mbeki called upon the UN member states to put into practice the Johannesburg Plan of Implementation that was adopted in 2002.^17^ In 2007, Brazilian President da Silva proposed the idea for a new conference on sustainable development and formed coalitions with the governments of developing countries.^17^ Support from different international forums such as the G20 paved the ground for the revival of the sustainable development agenda,^18^ which was designed at the Rio+20 Summit in 2012. Debates about the content and scope of a post‐MDG agenda—often called the post‐2015 agenda—facilitated the creation of the SDG agenda further.

Most remarkably, the delegations of Colombia and Guatemala put forward the idea for launching the SDGs.^19^ The two countries are not widely conceived of as leaders in the field of sustainable development.^20^ Their pledge was to pay more attention to climate change and environmental protection, thereby reviving the original conceptualization of sustainable development as defined by the Brundtland Commission of 1987.^21^ While the government of Brazil and multiple international scientific and political panels supported this proposal, the Europeans “were initially rather lukewarm.”^22^ As the European governments changed their position and became supportive of the proposal,^23^ in the outcome document “The Future We Want”, the heads of state and government and high‐level representatives established an intergovernmental process, the so‐called Open Working Group (OWG). The OWG was tasked with developing a set of global goals for sustainable development, which would then be agreed upon by the General Assembly in 2015 (e.g., ref. 24, see Note 2).

In the beginning, the different UN divisions had different ideas about how to design the 2030 Agenda for Sustainable Development. At the most general level, some divisions supported a human development oriented approach, whereas others were in favor of sustainable development. A series of events led to the convergence of the preferences within the UN to pursue human development and sustainable development in an integrated manner; these events include the global economic and financial crisis that unfolded in 2007/2008 and the recognition of the urgency of climate change.^25^ The OWG proposed 17 SDGs in mid‐2014,^4^ and in so doing it aimed to overcome compartmentalization by promoting integrated approaches to the economic, social, and environmental challenges confronting the world.^11^, ^15^, ^26^

At the 70th session of the UN General Assembly from September 25–27, 2015, the 2030 Agenda for Sustainable Development consisting of 17 SDGs, 169 associated targets, and 304 indicators was adopted after “three years of multistakeholder consultations and intergovernmental negotiations” (e.g., ref. 27, see Note 3). Three additional high‐level international meetings that took place before and after the UN Assembly in September 2015 shaped the design of the SDGs. The first of these meetings dealt with the question of financing the implementation of the SDGs and took place at the International Conference on Financing for Development held in Addis Ababa in July 2015. Second, the Turkish presidency during the G20 Summit in Alanya in November 2015 stressed the need for prioritizing the SDGs in international cooperation.^18^ Third, new climate goals for the post‐Kyoto climate regime were negotiated during the 21st Conference of the Parties (COP21) of the UN Framework Convention on Climate Change (UNFCCC) held in Paris in December 2015, which also had an impact on the design of the SDGs.^24^

Three features of the SDGs stand out if compared to previous understandings of global development, in particular the MDGs, of which all have implications for their implementation by the individual UN member states. First, universalism guides the implementation of the goals, that is, the goals shall be implemented by all states—rich and poor—that agreed on the 2030 Agenda.^8^ Second, the SDGs are “an integrated set of global priorities and objectives that are fundamentally interdependent.”^2^ This indivisibility of the 2030 Agenda requires a focus on policy coherence for effective implementation.^28^ Third, this goal‐ and target‐based policy framework requires careful monitoring and evaluation, which shall be part of the implementation process.^29^, ^30^ These three features are captured by the subsequent analysis of the national approaches to the implementation of the SDGs.

## 3 Analytical Perspectives on Policy Coherence and Integration

Policy coherence for sustainable development is enshrined in Target 17.14. As a concept, policy coherence is not an invention of the SDGs, but has been around for three decades in discourse among both practitioners and academics. The term was first used by the Development Assistance Committee in 1991 and was institutionalized through the publication of strategic documents by the European Commission and the OECD in the field of development policy throughout the 1990s and 2000s.^31^, ^32^ Conceptually, policy coherence is closely related to “policy integration” (e.g., refs. 13 and 33, 34, 35, 36, 37). Policy integration is also the term Le Blanc,^11^, ^15^ for example, uses in his empirical assessment of the SDGs. Likewise, the term “nexus” has been used to refer to a similar concept (e.g., refs. 38 and 39)—also in the context of the SDGs (e.g., ref. 17). The term “policy coherence” is mostly applied in studies of development policy, whereas “policy integration” is predominantly used by studies concentrating on climate and environmental policy, and “nexus” on the alignment of climate change, energy, food, and water policy.^13^ The latter often refers to policy domains that have a spatial dimension, for instance, when referring to land use or water resources (e.g., ref. 40).

The point of departure of the academic literature on policy coherence—and related concepts—is the well‐known pattern that governments tend to react to policy problems by adopting specialized policy measures, which could be an efficient approach as this allows for making use of policy expertise.^41^ The decision‐makers involved can rely on routines and the transaction costs of governing through specialized policies tend to be low. Although specialized policies can have an advantage to solve certain policy problems, they also have their limitations. In certain cases, this approach may even prevent the effective targeting of a given policy problem (e.g., ref. 42). Case in point is the adoption of two policies in two different policy domains that contradict each other, and hence undermine the attainment of the goals in one or even both of them. A rural development policy that promotes the production of biodiesel and therefore the cultivation of maize, may endanger an environmental policy that seeks to increase biological diversity.^43^ To circumvent the danger of different policy areas hampering each other, horizontal policy coordination and integration is needed.

Thus, at times, the need arises to coordinate the policies adopted in one policy field with the policies adopted in another field, which corresponds to a very basic definition of policy coherence.

The “going together of policies,” as May et al.^14^ put it, can happen in different ways and at all phases of the policy cycle. Health policy is an ideal case to illustrate the differences in pursuing policy coherence: First, non‐health policy fields can be encouraged to adopt policies that advance health objectives (intersectoral policies). For example, policies promoting electrification in rural areas—as one type of energy policy—can also help to improve the infrastructure for health services and therefore help to further SDG 3 on ensuring healthy lives and the promotion of wellbeing for all at all ages. When adopting this perspective, the evaluation of policy measures related to non‐health policy fields would need to consider their effects on the goal of promoting public health.

Second, instead of proposing field‐specific policies, the policy measures could be designed in such a fashion that they potentially attain objectives in health and other policy fields at the same time (multisectoral policies). For example, the introduction of school meals can help to attain the goal of promoting health and wellbeing along with attaining the goal of ensuring education (SDG 4).

The intersectoral goals/targets correspond to what Nilsson et al.^12^ refer to as “enabling” or “reinforcing” and the multisectoral ones to what they refer to as “indivisible.”

Drawing on the scientific literature and the conceptual debates therein, we are interested in how the states attempt to implement the SDGs in a fashion to attain policy coherence. As Persson et al.^29^ argue, arrangements for the implementation of the SDGs were discussed but not defined while they were negotiated, leading to a situation in which “guidance on national implementation arrangements is therefore sparse.”^7^, ^27^ Building on this assessment, we argue that the implementation of the universal and, thus, vaguely defined target of enhancing policy coherence will result in different implementation approaches. However, the way in which the individual SDGs are defined by the UN should at least offer some guidance on how to attain the goal of policy coherence when states implement the SDGs.

We reckon, in line with Peters,^41^ that the more integrated the SDGs are, the more they will entail substantive changes to policies and institutions. More generally, we differentiate between a substantive and a procedural approach to policy coherence. The substantive approach is about changing policy content and “goods and service production and delivery in society,” whereas the procedural approach is about altering the policy process only.^44^ We broaden the definition given by Howlett and Rayner^44^ of substantive policies to include institutional reforms necessary to deliver on policies. Substantial changes in the content of SDG policies would lead to an integration of several policies. Such complex changes in content are unlikely to be achieved without procedural and institutional changes. Our reasoning culminates in the following two sets of expectations.

- When the SDGs contain goals/targets that cut across the individual SDGs and aim for a mutual attainment of the goals, we expect national governments to announce procedural or institutional changes to policy‐making and implementation in order to attain policy coherence (intersectoral approach).
- When the SDGs encourage the adoption of joint goals and/or targets, we expect substantive policy changes in order to attain policy coherence (multisectoral approach). Substantive policy changes involve changes to the policy content and the corresponding institutional arrangements for their formulation and implementation.

## 4 Clarifications on Data and Methodological Procedure

This study concentrates on the SDGs addressing climate change (SDG 13), energy (SDG 7), agri‐food (SDG 2), health (SDG 3), and water provision (SDG 6). These areas were chosen due to their relevance to ongoing debates on how to attain sustainable development.^1^, ^39^, ^45^, ^46^ Originally, health was not part of this group of debates on interrelated global challenges (e.g., ref. 17), but it was added since it is increasingly recognized as an outcome of sustainable development, and not only a necessary precondition for it.^47^ In addition, recent studies about the interlinkages between individual SDGs show that the five goals studied here are amongst those with the strongest positive interconnection of the 2030 Agenda.^2^, ^15^

The following example illustrates how public health is affected by the policies primarily addressing climate change, energy, food, and water. Coal combustion stemming from the production of electricity is a large contributor to GHG emissions and to fine particulate air pollution. Policies that aim to reduce coal combustion can reduce mortality due to reducing fine particulate air pollution. A large proportion of child mortality is attributable to unsafe drinking water and inadequate sanitation (e.g., refs. 48 and 49). Freshwater resources are threatened by overuse, pollution, and climate change, which undermine access to clean drinking water and therefore increase the risk of water‐related disease.^47^ Climate change also has direct impacts on human health, stemming, for example, from global temperature increases (e.g., ref. 50). In this regard, Kickbusch^51^ states that the Paris agreement on climate change not only defines future action in this realm, but also in global health. Poor nutrition causes newborn and child deaths, while, conversely, more biodiverse agricultural production systems can help foster improved food security and improved nutritional status among rural populations.^47^

We are interested in examining how many and what types of interlinkages each of the five goals produce with the other SDGs since this informs our expectations regarding the national implementation approach. Le Blanc^11^, ^15^ has already demonstrated that the SDGs are designed in such a fashion as to create interlinkages between the individual goals and form a complex network of goals (e.g., ref. 10, see Note 4). Positive interactions between SDGs outweigh trade‐offs and counteracting effects between goals, which is favorable to facilitating policy coherence across policy domains. However, when assessing the five goals, trade‐offs must be taken into account because they might counter policy measures to reinforce integration.

In order to analyze the interlinkages between SDGs, we relied on qualitative content analysis and coded direct textual references to climate change, (renewable) energy, agri‐food, health, and water provision in the targets of the other SDGs. We sought to capture to what extent the targets of the SDGs are conducive to attaining SDG 2 (agri‐food), SDG 3 (health), SDG 6 (water), SDG 7 (energy), and SDG 13 (climate change). We base the selection of goals on previous studies, which conclude that there exist the strongest interlinkages (e.g., refs. 2 and 11, see Note 5). While these studies assess interlinkages between all goals, we focus on interlinkages between an individual goal and the targets of other goals.

Our coding approach consists of two steps (see **Table** **1**). First, we code targets where direct references are made to the aforementioned SDGs by referring to the final list of proposed SDGs.^52^ In a second step, we assign the form of interaction to each interlinkage by classifying them as either implying an intersectoral approach or multisectoral approach. An interlinkage is conceived as intersectoral if SDGs affect each other and are to be attained mutually, and as multisectoral if goals or targets shall be attained jointly.

**Table 1:** Policy coherence and forms of interlinkages between SDGs

	Forms of interlinkages between the SDGs	
Approaches to policy coherence	Intersectoral	Multisectoral
Procedural	e.g., creation of interministerial committees	e.g., institutional changes due to adoption of new policies
Substantive	e.g., adoption of new policies or reform of existing ones	e.g., adoption of new policies or reform of existing ones

Notes: Own elaboration based on Howlett and Rayner.^44^

The next step of the analysis shifts away from the definition of the SDGs to their implementation. Assessing UN member states' approaches to policy coherence in the Agenda 2030 is in the focus of the analysis. We analyze whether and how UN member states define the interlinkages between SDGs and intend to address policy coherence when implementing the SDGs. We focus on policy approaches and not the practice of SDG implementation because the period since the adoption of the SDGs in 2015 is too short and most states just started to define or implement SDG policies. We encourage future studies to address the on‐the‐ground implementation of the SDGs.

This analysis draws on the Voluntary National Reviews (VNRs) of the SDGs submitted in 2016 and 2017 and as reported by the Sustainable Development Knowledge Platform (see refs. 53 and 54, see Note 6). As policy integration not only depends on the willingness of policy‐makers to pursue it, but also depends on the capacity of their political and administrative systems, we selected countries belonging to different income groups to capture the variation in capacity (see Note 7). The literature has compellingly shown that the level of income is an adequate predictor of administrative or government capacity (e.g., ref. 55). Moreover, many authors argue that centralized agency and leadership is conducive to policy integration.^56^, ^57^ We thus used the following criteria that allow for gauging centralization in policy‐making: type of regime (authoritarian versus democratic), government system (presidential versus parliamentarian), and organization of state (centralized versus decentralized/federal) (see Note 8). Seeking for a high variation among the cases, we chose the following six countries: Benin, Colombia, Ethiopia, Qatar, Turkey, and Switzerland (see **Table** **2**).

**Table 2:** Countries selected for the analysis

Types	Least developed country	Middle income country	High income country
Centralized	Ethiopia	Turkey	Qatar
Decentralized	Benin	Colombia	Switzerland

Notes: Own elaboration.

While it is too early for an assessment of the implementation process, the analysis can still offer some indicative and preliminary findings. The SDG framework encourages member states to conduct regular progress reviews for meeting the goals, which are then presented to the annual High‐Level Political Forum at the UN in New York.^53^, ^54^ The regular reviews are voluntary and are meant to provide a platform for policy learning through sharing experiences in the implementation of the 2030 Agenda.

## 5 Empirical Assessment of the Links among the SDGs

In what follows, we analyze interlinkages between the five SDGs and all other SDGs except for SDG 16 and 17 as the latter two can be conceived as enablers rather than goals by themselves. None of the SDGs will be achieved in the absence of peace and effective governance (SDG 16). Material and institutional means for implementing the SDGs (SDG 17) are another precondition that must be met for the attainment of all SDGs.

The first SDG of relevance for this study is SDG 2 (Agri‐food), which seeks to end hunger, achieve food security and improved nutrition, and promote sustainable agriculture. SDG 2 is connected to targets associated with eight different SDGs. Food security and eradicating poverty (1.1. and 1.2) go hand‐in‐hand. Target 1.4 connects agri‐food governance to the right of owning and controlling land, which is an important precondition for practicing agriculture, whereas Target 5.a states this more specifically for women and girls. Maternal health, preventing death of newborns (3.1 and 3.2), and reducing communicable disease (3.3) are more likely to be achieved through better nutrition. Water quality (6.3) can be improved through sustainable agriculture, which reduces pollution. Sustainable investments in agriculture help to improve water‐related ecosystems (6.6). Increased biofuel production for renewable energy (7.2) can be the result of more agricultural productivity, which, in turn, can improve access to affordable energy services (7.1). Target 11.3 speaks to attaining SDG 2 by calling for sustainable urbanization, which takes into account land requirements for agriculture. Food production is addressed by Target 13.2 to the extent that it calls for integrating climate change measures into national policies, strategies, and planning in such a fashion that food production is not threatened (Indicator 13.2.1). In addition, resilient and adaptive agriculture systems (13.1 and 13.3) can help to improve adaptation to climate change. Lastly, Target 15.3 calls for combating desertification and restoring degraded land and soil, which again supports agricultural food production. Targets related to SDG 15 all relate closely to agricultural impacts on terrestrial ecosystems.

SDG 3 seeks to ensure health and well‐being for all and at every stage of life and is connected to six sets of targets. One of the key innovations of the SDGs is the incorporation of universal health coverage.^58^ While there are divergent conceptualizations over what progress toward universal health coverage means,^59^ it will require establishing or strengthening national arrangements for social protection so that it includes coverage of the poor and the vulnerable (Target 1.3). Target 2.1 is about ending hunger and ensuring access to sufficient food with a view to combat undernourishment, which is reiterated by Target 2.2, which emphasizes the nutritional needs of children and adolescents, pregnant and lactating women, and older persons. SDG 3 is also interlinked with three targets of SDG 5, which refers to ending all forms of violence (5.2) and harmful practices (5.3) against women and girls, and ensures universal access to sexual and reproductive health and reproductive rights (5.6). Target 6.1 is related to health as it calls for achieving universal and equitable access to safe and affordable drinking water, and Target 6.2 demands access to adequate and equitable sanitation and hygiene. There is a close relationship between increased health and well‐being of individuals and their contribution to economic growth (8.1) and their ability to work (8.5 and 8.6). In turn, economic growth can increase public health spending. Inclusive cities contribute to better health through better housing (11.1) because of reduced exposure to communicable diseases, and to natural hazards.

SDG 6 regards the availability and sustainable management of water and sanitation for all; this goal is connected to the targets of five other SDGs. The first connection exists with Target 1.4, which calls for ensuring that all men and women have access to basic services, including water and sanitation. Target 3.3 is about combating water‐borne diseases and Target 3.9 concerns the reduction of the number of deaths and illnesses from hazardous chemicals, and air, water, and soil pollution (see also Target 11.5). Access to safe and affordable drinking water is a precondition of sufficient nutrition (6.1 and 6.2). Target 12.4 approaches water issues from the perspective of the environmentally sound management of water resources, which aligns with Target 15.1 on the conservation, restoration, and sustainable use of terrestrial and inland freshwater ecosystems and their services.

SDG 7—access to affordable, reliable, sustainable, and modern energy for all—is connected to the targets of eight other SDGs. The first one is Target 1.4, which refers to access to basic services, including electricity. Bioenergy production can increase agricultural jobs and farm wages (2.1). Affordable energy and improved energy efficiency for agriculture can contribute to achieving increased food productivity (2.2) and indirectly improve food security (2.1). Automatic refrigeration is necessary for medicines and vaccines (3.8). More renewable energies will contribute to less air pollution (3.9) and energy‐saving measures such as cycling can improve the health of individuals (3.4). Improving energy efficiency and increasing renewable energies have mitigating effects on climate change (13). Access to affordable energy gives more time to individuals to go to school or work, thus improving education and employment (8.3, 8.5, and 8.6). Target 9.4 refers to the upgrading of infrastructure and retrofitting industries to make them sustainable, increasing resource‐use efficiency, and facilitating the adoption of clean and environmentally sound technologies and industrial processes. Target 12.c is about rationalizing and phasing‐out fossil‐fuel subsidies. Target 13.2 is about integrating climate change with other sectoral policies, which includes the integration of climate and energy policy.

Climate change is the theme that is incorporated in the greatest number of other SDGs, nine of them to be specific. Target 1.5 calls for building the resilience of the poor and those in vulnerable situations and to reduce their exposure and vulnerability to climate‐related extreme events. Target 2.4 indicates that food production and agricultural practices must be modified to strengthen the capacity for adaptation to climate change. Additionally, raising awareness for climate change can foster sustainable agriculture systems. There are interlinkages between combatting climate change and improving public health (3.4, 3.8. and 3.9). All five targets related to energy production concern actions necessary to combat climate change since deep decarbonization of the electricity sector is necessary to eventually attain SDG 13.^60^, ^61^, ^62^ Similar to attaining SDG 7 on energy governance, the achievement of the climate‐related goals is interlinked with Target 9.4 on the upgrading of infrastructure and increasing the resource‐use efficiency of industry. Economic growth can negatively affect the environment, in particular air, water, and soil pollution (8.1). Target 11.b is about increasing the number of cities and human settlements adopting and implementing integrated policies and plans for the mitigation of and adaptation to climate change. Targets 14.1–14.6 are strongly interlinked with combating climate change because they refer to improved coastal and marine ecosystems. Target 15.2 concerns the implementation of sustainable forest management, the halt of deforestation, the restoration of degraded forests, and increasing afforestation and reforestation.

The interlinkages of the five themes with the targets of the other SGDs are summarized in **Table** **3**. Climate change with 9 interlinkages is closely followed by agri‐food and energy with 8 goals. Fewer connections can be observed for health (6) and water (5). With regards to the types of interlinkages established by the SDGs, Table 3 also indicates which targets/goals are linked to one another in an intersectoral (I) and a multisectoral (M) way. While overall the intersectoral interlinkages clearly dominate, it is worth pointing out that in the thematic areas of water and climate governance, the multisectoral approach appears to be more important. Consequently, we expect national governments to be more likely to propose substantive approaches to integrate the SDGs on water and climate governance compared to the other three SDGs of interest here. More broadly, we interpret this finding as one of the ways in which the SDGs provide guidance on national implementation arrangements.^7^, ^27^, ^29^

**Table 3:** Links between the five themes and the other SDGs

SDGs	SDG2: Agri‐food	SDG 3: Health	SDG 6: Water	SDG 7: Energy	SDG 13: Climate
1. Poverty	1.1; 1.2, 1.4 (I)	1.3 (M)	1.4 (M)	1.4 (I)	1.5 (M)
2. Agri‐food		2.1, 2.2, 2.3 (I)		2.1 (I)	2.4 (M)
3. Health	3.1, 3.2., 3.3 (I)		3.3, 3.9 (M)	3.4, 3.8, 3.9 (I)	3.4, 3.8, 3.9 (I)
4. Education					
5. Gender equality	5.a and b (I)	5.2, 5.3, 5.6 (I)			
6. Water	6.1, 6.2, 6.3 (I) 6.6 (M)	6.1, 6.2 (I)			
7. Energy	7.1, 7.2 (I)				7.1–3, 7.a–b (M)
8. Growth		8.1, 8.5, 8.6 (I)		8.3, 8.5, 8.6 (I)	
9. Infrastructure				9.4 (I)	9.4 (I)
10. Inequality					
11. Cities	11.3 (I)	11.1 (M)	11.5 (M)		11.b (M)
12. Consumers			12.4 (M)	12.c (I)	
13. Climate	13.1 (M); 13.2, 13.3 (I)			13.2 (I)	
14. Maritime life					14.1–6 (M)
15. Land life	15.1, 15.2, 15.3, 15.4 (I)		15.1 (M)		15.2 (M)
Number of goals	8	6	5	7	9
Number of types	8 (I) 2 (M)	4 (I) 2 (M)	5 (M)	7 (I)	2 (I) 6 (M)

Notes: Own elaboration. I = intersectoral approach; M = multisectoral approach.

## 6 National Implementation Approaches toward Policy Coherence

In this section, we examine the VNRs submitted in 2016 and 2017 by Benin and Ethiopia, Colombia and Turkey, and Qatar and Switzerland. Three sets of questions guide the analysis of the country cases. First, do governments define priorities for the implementation of the SDGs? Second, do governments—implicitly or explicitly—classify the interlinkages between the SDGs as intersectoral or multisectoral? Third, do governments propose a substantive or procedural approach for implementing the SDGs?

### 6.1 Low Income and Least Developed Countries: Benin and Ethiopia

Ethiopia acknowledges its commitment to the SDGs but does not refer to the interlinkages between individual SDGs.^63^ The VNR sets out to achieve the specific targets by adopting a “silo” approach, which is the opposite of policy coherence. One reason for this might be that Ethiopia generally pursues a strictly sectoral approach for solving policy problems.^64^ According to the government, the SDGs are fully integrated in Ethiopia's Second Growth and Transformation Plan (GTP II), which was adopted in parallel with the SDG process. The SDG targets that meet the national priorities of the GTP II are integrated into the national and regional sectoral policies.^63^

Ethiopia's development priorities focus on agricultural sector development, structural economic transformation and productivity, urbanization, and anticorruption policies.^64^ In the GTP II, single SDG targets—although not named explicitly—refer to the overarching priorities and goals of the SDGs. For example, efficient and clean energy (SDG 7) is recognized as a precondition for sustainable agriculture (SDG 2), while clean and affordable water (SDG 6) is stated to foster agricultural sector development (SDG 2). The government has adopted an intersectoral strategy for a “climate resilient economy,” which integrates SDGs 2 (agri‐food), 6 (water), 7 (energy), and 13 (climate).^63^

The Ethiopian government adopts a mostly sectoral and punctually intersectoral strategy for selected SDGs for attaining policy coherence. Synergies and trade‐offs between SDGs are not explicitly mentioned but the report states that the individual SDGs will affect each other.^63^ The government emphasizes that the SDGs are fully integrated in the GTP II and, thus, are intended to be the subject of future substantive changes in national policies and institutions. The Ethiopian Planning Commission argues that implementing the SDGs is legally binding in Ethiopia because the Council of Ministers and the House of Peoples' Representatives adopted the “SDG‐integrated GTP II” in 2016.^63^ At the same time, it is evident that the GTP II is the guiding policy document and not the SDGs. The SDGs are supposed to become part of substantive policy changes in Ethiopia, which include the implementation of a decentralized administrative system.

Benin's VNR reveals the opposite approach to the Ethiopian government.^65^ First of all, timing was a crucial factor that allowed Benin to fully endorse the SDGs in its National Development Plan.^65^ This allowed the government to organize a needs assessment with a wide range of stakeholders. As a result of this process, 49 SDG targets were identified as priorities for the future National Development Plan of Benin. All goals and their interlinkages of the present analysis were considered in Benin's list of priorities. It stands out that they refer less to structural changes of the economy or society—as was the case for Ethiopia—but more to targets where access of individuals to certain services (e.g., clean water, affordable energy) are highlighted. The VNR further underlines the relevance of climate change (SDG 13) because of its massive impacts on agri‐food, water, health, infrastructure, energy, and environment.^65^ The government thus integrates the SDGs and the Paris Agreement by conceiving the National Plan for Adaptation to Climate Change as a “transversal” plan for achieving the SDGs.

Both, an inter‐ and multisectoral understanding of the interlinkages between SDGs emerge from the VNR. The majority of the VNR (pp. 16–37) is dedicated to the analysis of the synergies and trade‐offs between the 49 targets. Although the analysis focuses on the mutual effects of SDG targets (intersectoral), it also highlights the need for mutually attaining a specific goal through various sectors, climate change (multisectoral) in particular. Based on its analysis, the government stresses the need for creating synergies between sectors through at least an intersectoral approach.^65^ Whether a more integrated approach will be taken (multisectoral) depends on the next steps of policy formulation. The government expects to finalize its new National Development Plan in 2017.

Benin has pursued a procedural approach so far. The government established a new committee and new procedures for coordinating and monitoring SDG implementation. This committee is composed of actors from the government, public administration, civil society, labor unions, international donors, and business. Technical units that oversee the implementation of targets complement the committee. Political relevance and effectiveness shall be granted through a high‐level composition of the committee, which shall report to the Minister of State of the Planning and Development Ministry. Substantial changes will depend on the still‐to‐be‐formulated new National Development Plan. However, given the high integration of SDGs in the government's VNR, substantial changes are likely to take place.

### 6.2 Middle Income Countries: Colombia and Turkey

The Colombian government embraced a particularly ambitious approach to the attainment of the SDGs, which it conceived as a process that takes place simultaneously with the internal peace‐building after a period of long civil war, accession to the OECD, and the implementation of the country's green growth strategy.^66^ Being one of the governments that pushed for the adoption of the SDGs,^19^ Colombian policy‐makers started to implement the SDGs in the context of its National Development Plan 2014–2018.^66^ Similar to Ethiopia, the government sets out a green growth strategy in order to attain SDGs 2, 6, 7, and 12–15.^67^ While the government is committed to the SDGs as a whole, it has defined poverty (SDG 2), health (SDG 3), economic growth (SDG 8), climate change (SDG 13), and partnerships for the goals (SDG 17) as priority areas.^68^

The Colombian government undertook an assessment of the extent to which subnational and local governments consider the SDGs in their development plans. This assessment revealed that there are marked regional disparities, which induced the government to make the vertical coordination of policy efforts a priority area.^66^ The subnational level is important in the Colombian context since the government differentiates between the national and the subnational level for integrating policies. According to the VNR, the national level is responsible for the attainment of SDGs 4–7, whereas the subnational level is responsible for 1, 2, 4, 5, and 11. For the SDGs 10, 14, and 17, the responsibility is assigned to the international level, and for 8, 9, and 12, the private sector is assigned the competence to attain their realization.^67^

We can state that there is a clear orientation towards an approach to attain substantive policy change, which includes new procedures to attain policy coherence. An important step is the establishment of the “High Level Commission for Effective Implementation of the 2030 Agenda and the SDGs” in February 2015. The commission draws on information supplied by intersectoral working groups,^67^ which suggests that the overall approach to the implementation of the SDGs is likely to be an intersectoral one. On specific topics, however, the government embraces a multisectoral approach; this holds particularly true for the green growth strategy.

As the UN (2016)^66^ stresses in its synthesis report, the country has established institutional mechanisms at the highest level of government that bring leadership into the implementation process. Furthermore, a cross‐parliamentary group participates in monitoring the attainment of the goals and targets, and the government collaborates with the municipal and departmental authorities to disseminate the SDGs and to develop adequate policies for local development.

The VNR submitted by the government of Turkey indicates that the government is committed to implementing all 17 SDGs; that is, it does not define any priorities for the implementation process. Rather, the Turkish government regards the implementation of the SDGs to correspond to the attainment of sustainable development as it was defined at the 1992 Rio Conference. From this, it follows that the individual SDGs are not the subject of policy coherence, but more broadly the mutual attainment of economic, ecological, and social sustainability.^69^ This is an interesting observation since the Turkish government has been committed to this broader understanding of sustainable development since the publication of its 7th National Development Plan in 1996.^66^ In other words, the Turkish approach is one where the SDGs are interpreted in the same ways as previous attempts to attain sustainable development, indicating that the government reiterates the policy approaches it has taken in the past. This interpretation is supported by the UN synthesis report, which states that the country aims to continue its best practice examples.^66^

According to the information presented by the Sustainable Development Knowledge Platform,^70^ the Turkish government argues that there is a high coherence between its 10th Development Plan and the SDGs. Nonetheless, the government announced that it would integrate the SDGs even more systematically in its 11th Development Plan,^66^ which is currently being developed. Despite its commitment to an encompassing implementation approach and recognition that “breaking silos and working together on particular goals in an integrated manner will be a critical challenge,”^69^ the Turkish report does not discuss links between the individual SDGs in detail. In marked contrast to the previous VNRs analyzed, there is also no emphasis placed on fighting climate change.

Thus, no reference is made in the relevant documents to adopting either an inter‐ or multi‐sectoral approach to implement the SDGs. The Turkish government asserts that “the best ways to integrate sustainable development policy‐making at all levels and the opportunities for or barriers to integrating the three dimensions of sustainable development will be explored.”^70^ It further stresses the use of a procedural approach while keeping the substance of its policies as set out in the 10th National Development Plan.^69^ The Ministry of Development along with the Sustainable Development Coordination Commission will coordinate the implementation of the SDGs, but all ministries will be involved in delivering on them.^66^

### 6.3 High Income Countries: Qatar and Switzerland

The government of Qatar emphasizes that its understanding of sustainable development comprises human, social, economic, and environmental development.^71^ SDG implementation is to be attained by the 2nd National Development Strategy (2017–2022), which prioritizes eight sectors: education and training (SDG 4), health care (SDG 3), social protection (SDGs 1, 2, and 5), cultural enrichment and sports excellence (SDGs 3 and 17), public security and safety (SDGs 11 and 16), international cooperation (SDGs 10, 12, and 17), economic diversification and private sector development (SDGs 2, 6, 8, 9, and 12), and environmental sustainability, natural resources, and economic infrastructure (SDGs 6, 7, and 11–15). Remarkably, climate change is not treated as a specific priority area, but rather as only one dimension of the clustered set of SDGs on environmental sustainability.

The strategic document submitted by the government of Qatar maps the priorities of the National Development Strategy on the SDGs. Two areas (education and training, and health care) are not linked with other sectors, whereas the great majority of the priority areas are marked by several interlinkages to other goals. While this is not mentioned explicitly in the document, it appears likely that the attainment of the National Development Strategy and therefore the SDGs will be accompanied by the adoption of a substantive approach. This expectation is derived from the fact that the government identifies and acknowledges numerous interlinkages and discusses their implications for policy‐making. Regarding the procedures to attain the SDGs, the report assigns one or several ministries to the eight thematic areas outlined above, but it is not explained how they are expected to cooperate with one another. On the basis of the empirical material available at this point in time, it is difficult to judge whether the implementation of the SDGs will follow an inter‐ or multisectoral logic. The VNR submitted by the country, however, contains an explicit commitment to attaining policy integration.^72^

The Swiss government conceives of the SDGs as thematic clusters that need to be addressed jointly in order to attain their underlying policy goals:^73^ Consumption and production (SDG 12), urban development, mobility and infrastructure (SDGs 9 and 11), energy and climate (SDGs 7 and 13), natural resources (SDGs 2, 6, 14, and 15), economic and financial system (SDGs 8, 10, 16, and 17), education, research and innovation (SDG 4), social security (SDGs 1 and 16), social cohesion and gender equality (SDGs 5, 10, and 16), and health (SDG 3).

The approach adopted by the government of Switzerland is an interesting one since the clusters formed do not correspond to the interlinkages we identified when consulting the SDGs directly. As stated above, we found that climate change has the greatest number of linkages with other thematic areas (see Table 3). When looking at the Swiss approach, the governance of natural resources, for example, is associated with more SGDs than climate governance. A second observation worth noting is that the identification of thematic clusters is likely to result in the adoption of a multisectoral approach since the Swiss strategic document summarizes the goals and makes their attainment less likely by pursing an intersectoral approach. Yet similar to some other states, the Swiss government understands the realization of the SDGs as a realization of sustainable development as defined 1992 at the Rio Earth Summit. From this perspective, the Swiss and the Turkish perspective on sustainable development are similar.

As reported by the Sustainable Development Knowledge Platform,^74^ the Swiss national implementation strategy spells out that a substantive approach is needed, that is, that policies have to become modified to attain the SDGs. The Swiss government explicitly connects the realization of the SDGs with the design of its foreign policy, including foreign economic policy, international cooperation, and sectoral foreign policies. In addition to the policy dimension, the Swiss strategy contains a clearly discernible procedural approach that concerns the modification of institutional arrangements such that they can facilitate and support the coordination of national, subnational, and international processes.^74^ Similar to most of the previous VRNs, it is difficult to judge whether an inter‐ or multisectoral approach will be adopted by the Swiss government to attain policy coherence when implementing the SDGs. Considering the strong reputation Switzerland has in implementing policies related to sustainable development,^75^ this is an indication that the question of whether an inter‐ or multisectoral approach is embraced by the national government is premature.

### 6.4 Insights from the Case Studies

Altogether, we can conclude that the six countries analyzed have indeed adopted different approaches to the implementation of the SDGs (see **Table** **4**). Nonetheless, when abstracting away from the details of the national implementation approaches, we can identify some common patterns. First, four of the six countries defined priority areas whereas Switzerland and Turkey abstained from doing so. Second, with the exception of Turkey, the VNRs analyzed suggest that both an inter‐ and a multisectoral approach will be adopted to attain policy coherence. In the cases of Colombia and Ethiopia, the multisectoral approach is limited to the attainment of green growth. Drawing on their VNRs, the other three countries seem to consider applying both approaches to a broader set of SDGs. From this, it follows that there is more cross‐country variation than variation between the individual SDGs. For the latter to hold true, we would have needed to observe that climate and water governance is more often the subject of a multisectoral approach than the other SDGs of interest in this study.

**Table 4:** Different approaches of countries in SDG implementation

	Centralized authority			Decentralized authority		
	Ethiopia	Turkey	Qatar	Benin	Colombia	Switzerland
Priority areas	X		X	X	X	
Integration approach	Intersectoral, but selectively multisectoral	Intersectoral	Inter‐ and multisectoral	Intersectoral and multisectoral	Intersectoral, but selectively multisectoral	Inter‐ and multisectoral
Policy approach	Procedural and substantive	Procedural	Procedural and substantive	Procedural and substantive	Procedural and substantive	Procedural and substantive

Notes: Own elaboration.

The third dimension refers to the type of policy change indicated in the VNRs and here again Turkey is the only country that we interpret to pursue a strictly procedural approach. For the other countries, we found hints in the relevant documents that both procedural and substantive policy change is likely to be pursued by the respective governments.

Once we systematize the implementation approaches adopted by the individual member states, we cannot find any clear‐cut differences between them that would correspond to our theoretical reasoning. Therefore, we cannot support the expectation that the way in which the SDGs relate to each other produce specific approaches to their implementation. However, what is worth noting is that we could not find any patterns that would allow for differentiating between countries on the basis of their income level or the level to which political power is centralized or decentralized.

## 7 Conclusion

How do the SDGs related to food, water, and energy security as well as to health and the societies' capacity to mitigate and adapt to climate change relate to each other? How do national governments interpret the call for enhancing policy coherence for sustainable development? What are the implications of the respective national approaches for attaining the SDGs related to climate change, energy, agri‐food, health, and water provision? These three research questions guided this analysis.

The findings show that the governance of water and climate change are the themes that are best connected to other SDGs and should therefore be addressed by policy‐makers in the most coherent fashion, that is, by adopting a multisectoral approach to policy integration. While Colombia and Ethiopia indeed adopted multisectoral approaches to realize green growth and to address climate change in this way, the other countries have not adopted such an approach. Moreover, we could not identify the adoption of a multisectoral approach to water governance, which falsifies our theoretical expectations.

Turning to how the governments interpreted the calls to enhance policy coherence for sustainable development, the analysis of the VNRs submitted revealed quite some differences. Most importantly, some countries seem to interpret this call as one to attain policy coherence between the individual goals, whereas others concentrate on the individual dimensions of the respective goals and elaborate on how these can be attained at the same time. Moreover, some countries evaluate the goals with the broader concept of sustainable development. To be fair, all these interpretations are valid given that the SDGs do two things: First, they create links between the individual SDGs; second, they then separately state that policy coherence for sustainable development should be enhanced. It is understandable that the countries came up with different interpretations in their first implementation reports. The five SDGs we selected did not turn out to enjoy a privileged status in the VNRs.

We have shown that policy integration is a necessary condition for successfully implementing the 2030 Agenda. Dense and different types of interlinkages between SDGs require different procedural and institutional changes in the implementing countries. Our empirical analysis indicates that countries generally acknowledge the need to adopt and change their procedures and institutions in order to implement the SDGs. This is regardless of their assessment of the types of interlinkages between SDGs. Some countries do not classify the interlinkages at all. We can thus see no systematic relationship between the assessment of interlinkages by the implementing countries and their approaches for procedural and institutional change. This has implications for policy‐making. It is likely that neglecting the relationship between types of interlinkages and the type of procedural and institutional reform required for integrated SDG implementation will limit policy integration, which will not only concern the five SDGs analyzed here, but the entirety of the SDGs.

A question we did not ask here is whether policy coherence will really yield better policy results, which is arguably a research question we can only address when the implementation of the SDGs has progressed. However, a research question that other studies can elaborate on now is how various intergovernmental organizations position themselves on the attainment of the SDGs and whether and how they position themselves on the attainment of policy coherence in general and with regard to their respective thematic areas. Therefore, we must clearly acknowledge that an important limitation of our study is that we cannot offer an empirical assessment of global action. From this perspective, our study could be complemented by analyses of the positions of the UN Food and Agriculture Organization (agri‐food), the World Health Organization (health), the UN Environment Program (water), the International Energy Agency (energy), and International Renewable Energy Agency (energy), and the Secretariat of the UNFCCC (climate change). This group of organizations could be complemented by the European Union (EU) and the OECD, bringing in the position of a supranational organization (EU) and of an organization that represents the economically most developed states (OECD).

A second evident limitation is that this study is based on the plans and intentions of the individual countries to pursue policy integration when implementing the SDGs. Given the short period since the adoption of the SDGs in 2015, we cannot yet draw any conclusion about the actual levels of policy integration. Therefore, we invite future research to shift the analytical focus to policy coherence in practice at the national level.

## Notes

1. This focus on sustainability in the post‐MDG era was a result of the UN Conference on Sustainable Development—or Rio+20—held in Rio de Janeiro in 2012.
2. The Open Working Group on Sustainable Development Goals consisted of 70 countries. For details, see the following website: https://sustainabledevelopment.un.org/owg.html.
3. The 17 SDGs are (SDGs selected for the purpose of this study in italics): SDG 1: End poverty in all its forms everywhere (7 targets; 12 indicators); SDG 2: End hunger, achieve food security and improved nutrition and promote sustainable development (8 targets; 14 indicators); SDG 3: Ensure healthy lives and promote well‐being for all at all ages (13 targets; 26 indicators); SDG 4: Ensure inclusive and equitable quality education and promote lifelong learning opportunities for all (10 targets; 11 indicators); SDG 5: Achieve gender equality and empower all women and girls (9 targets; 14 indicators); SDG 6: Ensure availability and sustainable management of water and sanitation for all (8 targets; 11 indicators); SDG 7: Ensure access to affordable, reliable, sustainable, and modern energy for all (5 targets; 5 indicators); SDG 8: Promote sustained, inclusive, and sustainable economic growth, full and productive employment, and decent work for all (12 goals; 17 indicators); SDG 9: Build resilient infrastructure, promote inclusive and sustainable industrialization and foster innovation (8 targets; 12 indicators); SDG 10: Reduce inequality within and among countries (10 targets; 11 indicators); SDG 11: Make cities and human settlements inclusive, safe, resilient, and sustainable (10 targets; 15 indicators); SDG 12: Ensure sustainable consumption and production patterns (11 targets; 13 indicators); SDG 13: Take urgent action to combat climate change and its impacts (5 targets; 7 indicators); SDG 14: Conserve and sustainably use the oceans, seas, and marine resources for sustainable development (10 targets; 10 indicators); SDG 15: Protect, restore, and promote sustainable use of terrestrial ecosystems, sustainably manage forests, combat desertification, and halt and reverse land degradation, and halt biodiversity loss (12 targets; 14 indicators); SDG 16: Promote peaceful and inclusive societies for sustainable development, provide access to justice for all, and build effective, accountable, and inclusive institutions at all level (12 targets; 23 indicators); SDG 17: Strengthen the means of implementation and revitalize the Global Partnership for Sustainable Development (19 targets; 25 indicators).
4. According to Nilsson et al.^12^ there can be positive interactions between goals/targets that range from enabling, to reinforcing and even indivisible as well as negative ones where goals/targets constrain, counteract or at worst cancel one another out. If the interaction between the goals/targets is neither significantly positive nor negative, the authors regard them as being consistent. However, for the purpose of this analysis, we go with the differentiation between intersectoral and multisectoral policies and leave the empirical application of the conceptually compelling categorization to future research.
5. In addition, these studies find a dense network of interlinkages with SDG 10 (inequality) and SDG 16 (peace, justice, and governance) and other goals. Both SDG 10 and 16 are often conceived as preconditions for achieving other goals.
6. The Voluntary National Reviews focus on a specific set of SDGs each year (e.g., SDG 1, 2, 3, 5, 9, 14 in 2017). They are submitted to the Annual High Level Panel Forum at the UN.
7. We use four indicators to represent income groups of countries that are based on the World Bank's categorization of income and the United Nations' classification of least developed countries: Least Developed Country; Lower Middle Income Country and Upper Middle Income Country; High Income Country.
8. We use the following indicators: (a) classification of regime type by Varieties of Democracy, namely, closed autocracy, electoral autocracy, electoral democracy, and liberal democracy;^76^ (b) government system as outlined in the Constitution of each country, cross‐checked with the CIA Factbook; (c) organization of state as outlined in the Constitution of each country, cross‐checked with the CIA Factbook. These criteria are on a continuum between highly centralized (closed autocracy, presidential/theocracy/monarchy, central state) to decentralized policy‐making (liberal democracy, parliamentarian, federal).

## Conflict of Interest

The authors declare no conflict of interest.
